# Early non-disabling relapses are important predictors of disability accumulation in people with relapsing-remitting multiple sclerosis

**DOI:** 10.1177/13524585231151951

**Published:** 2023-02-27

**Authors:** Cyrus Daruwalla, Vahid Shaygannejad, Serkan Ozakbas, Eva Kubala Havrdova, Dana Horakova, Raed Alroughani, Cavit Boz, Francesco Patti, Marco Onofrj, Alessandra Lugaresi, Sara Eichau, Marc Girard, Alexandre Prat, Pierre Duquette, Bassem Yamout, Samia J Khoury, Seyed Aidin Sajedi, Recai Turkoglu, Ayse Altintas, Olga Skibina, Katherine Buzzard, Pierre Grammond, Rana Karabudak, Anneke van der Walt, Helmut Butzkueven, Davide Maimone, Jeannette Lechner-Scott, Aysun Soysal, Nevin John, Julie Prevost, Daniele Spitaleri, Cristina Ramo-Tello, Oliver Gerlach, Gerardo Iuliano, Matteo Foschi, Radek Ampapa, Vincent van Pesch, Michael Barnett, Nevin Shalaby, Marie D’hooghe, Jens Kuhle, Maria Jose Sa, Marzena Fabis-Pedrini, Allan Kermode, Saloua Mrabet, Riadh Gouider, Suzanne Hodgkinson, Guy Laureys, Liesbeth Van Hijfte, Richard Macdonell, Celia Oreja-Guevara, Edgardo Cristiano, Pamela McCombe, Jose Luis Sanchez-Menoyo, Bhim Singhal, Yolanda Blanco, Stella Hughes, Justin Garber, Claudio Solaro, Chris McGuigan, Bruce Taylor, Koen de Gans, Mario Habek, Abdullah Al-Asmi, Simu Mihaela, Tamara Castillo Triviño, Talal Al-Harbi, Juan Ignacio Rojas, Orla Gray, Dheeraj Khurana, Bart Van Wijmeersch, Nikolaos Grigoriadis, Jihad Inshasi, Jiwon Oh, Eduardo Aguera-Morales, Yara Fragoso, Fraser Moore, Cameron Shaw, Seyed Mohammad Baghbanian, Neil Shuey, Barbara Willekens, Todd A Hardy, Danny Decoo, Angel Perez sempere, Deborah Field, Ray Wynford-Thomas, Nick G Cunniffe, Izanne Roos, Charles B Malpas, Alasdair J Coles, Tomas Kalincik, J William L Brown

**Affiliations:** Department of Clinical Neurosciences, University of Cambridge, Cambridge, UK; Isfahan University of Medical Sciences, Isfahan, Iran; Dokuz Eylul University, Izmir, Turkey; Department of Neurology and Center of Clinical Neuroscience, First Faculty of Medicine, Charles University in Prague and General University Hospital, Prague, Czech Republic; Department of Neurology and Center of Clinical Neuroscience, First Faculty of Medicine, Charles University in Prague and General University Hospital, Prague, Czech Republic; Division of Neurology, Department of Medicine, Amiri Hospital, Sharq, Kuwait; KTU Medical Faculty Farabi Hospital, Trabzon, Turkey; Department of Medical and Surgical Sciences and Advanced Technologies, GF Ingrassia, Catania, Italy Multiple Sclerosis Center, University of Catania, Catania, Italy; Department of Neuroscience, Imaging and Clinical Sciences, University G. D’Annunzio, Chieti, Italy; Dipartimento di Scienze Biomediche e Neuromotorie, Università di Bologna, Bologna, Italy IRCCS Istituto delle Scienze Neurologiche di Bologna, Bologna, Italy; Hospital Universitario Virgen Macarena, Sevilla, Spain; CHUM and Universite de Montreal, Montreal, QC, Canada; CHUM and Universite de Montreal, Montreal, QC, Canada; CHUM and Universite de Montreal, Montreal, QC, Canada; Nehme and Therese Tohme Multiple Sclerosis Center, American University of Beirut Medical Center, Beirut, Lebanon; Nehme and Therese Tohme Multiple Sclerosis Center, American University of Beirut Medical Center, Beirut, Lebanon; Department of Neurology, Neuroscience Research Center, Golestan University of Medical Sciences, Gorgan, Iran; Haydarpasa Numune Training and Research Hospital, Istanbul, Turkey; Department of Neurology, School of Medicine and Koc University Research Center for Translational Medicine (KUTTAM), Koc University, Istanbul, Turkey; Department of Neurology, Box Hill Hospital, Melbourne, VIC, Australia Department of Neuroscience, Central Clinical School, Monash University, Melbourne, VIC, Australia Department of Neurology, The Alfred Hospital, Melbourne, VIC, Australia; Department of Neurology, Box Hill Hospital, Melbourne, VIC, Australia Department of Neuroscience, Central Clinical School, Monash University, Melbourne, VIC, Australia MS Centre, Royal Melbourne Hospital, Melbourne, VIC, Australia; CISSS Chaudière-Appalache, Levis, QC, Canada; Hacettepe University, Ankara, Turkey; Department of Neuroscience, Central Clinical School, Monash University, Melbourne, VIC, Australia Department of Neurology, The Alfred Hospital, Melbourne, VIC, Australia; Department of Neuroscience, Central Clinical School, Monash University, Melbourne, VIC, Australia; Centro Sclerosi Multipla, UOC Neurologia, ARNAS Garibaldi, Catania, Italy; School of Medicine and Public Health, University Newcastle, Newcastle, NSW, Australia Department of Neurology, John Hunter Hospital, Hunter New England Health, Newcastle, NSW, Australia; Bakirkoy Education and Research Hospital for Psychiatric and Neurological Diseases, Istanbul, Turkey; Monash Medical Centre, Melbourne, VIC, Australia Department of Medicine, School of Clinical Sciences, Monash University, Melbourne, VIC, Australia; CSSS Saint-Jérôme, Saint-Jerome, QC, Canada; Azienda Ospedaliera di Rilievo Nazionale San Giuseppe Moscati Avellino, Avellino, Italy; Hospital Germans Trias i Pujol, Badalona, Spain; Academic MS Center Zuyderland, Department of Neurology, Zuyderland Medical Center, Sittard-Geleen, The Netherlands School for Mental Health and Neuroscience, Maastricht University, Maastricht, The Netherlands; Ospedali Riuniti di Salerno, Salerno, Italy; Department of Neuroscience, Neurology Unit, S. Maria delle Croci Hospital of Ravenna, AUSL Romagna, Ravenna, Italy; Nemocnice Jihlava, Jihlava, Czech Republic; Cliniques Universitaires Saint-Luc, Brussels, Belgium Université Catholique de Louvain, Ottignies-Louvain-la-Neuve, Belgium; Brain and Mind Centre, Sydney, NSW, Australia; Neurology, Kasr Al Ainy MS Research Unit (KAMSU), Cairo, Egypt; Department of Neurology, National MS Center, Melsbroek, Belgium; Neurology, MS Center and Research Center for Clinical Neuroimmunology and Neuroscience Basel (RC2NB), Departments of Head, Spine and Neuromedicine, Biomedicine and Clinical Research, University Hospital Basel, University of Basel, Basel, Switzerland; Department of Neurology, Centro Hospitalar Universitario de Sao Joao, Porto, Portugal Faculty of Health Sciences, University Fernando Pessoa, Porto, Portugal; Perron Institute for Neurological and Translational Science, University of Western Australia, Nedlands, WA, Australia Centre for Molecular Medicine and Innovative Therapeutics, Murdoch University, Perth, WA, Australia; Perron Institute for Neurological and Translational Science, University of Western Australia, Nedlands, WA, Australia Institute of Immunology and Infectious Diseases, Murdoch University, Perth, WA, Australia Sir Charles Gairdner Hospital, Nedlands, WA, Australia; Department of Neurology, University Hospital Razi – Manouba, Tunis, Tunisia Faculty of Medicine of Tunis, University of Tunis El Manar, Tunis, Tunisia; Department of Neurology, University Hospital Razi – Manouba, Tunis, Tunisia; Immune Tolerance Laboratory, Ingham Institute and Department of Medicine, University of New South Wales (UNSW), Sydney, NSW, Australia; Department of Neurology, University Hospital Ghent, Ghent, Belgium; Department of Neurology, University Hospital Ghent, Ghent, Belgium; Austin Health, Melbourne, VIC, Australia; Department of Neurology, Hospital Clinico San Carlos, Madrid, Spain; Centro de Esclerosis Múltiple de Buenos Aires (CEMBA), Buenos Aires, Argentina; The University of Queensland, Brisbane, QLD, Australia Royal Brisbane and Women’s Hospital, Herston, QLD, Australia; Hospital de Galdakao-Usansolo, Galdakao, Spain; Bombay Hospital Institute of Medical Sciences, Mumbai, India; Center of Neuroimmunology, Service of Neurology, Hospital Clinic de Barcelona, Barcelona, Spain; Royal Victoria Hospital, Belfast, UK; Westmead Hospital, Sydney, NSW, Australia; Department of Neurology, ASL3 Genovese, Genova, Italy Department of Rehabilitation, M.L. Novarese Hospital, Moncrivello, Italy; St. Vincent’s University Hospital, Dublin, Ireland; Royal Hobart Hospital, Hobart, TAS, Australia; Groene Hart Ziekenhuis, Gouda, The Netherlands; Department of Neurology, University Hospital Center Zagreb, Zagreb, Croatia University of Zagreb, School of Medicine, Zagreb, Croatia; College of Medicine & Health Sciences and Sultan Qaboos University Hospital, Sultan Qaboos University, Seeb, Oman; Department of Neurology, Victor Babes University of Medicine and Pharmacy, Timișoara, Romania; Hospital Universitario Donostia and IIS Biodonostia, San Sebastián, Spain; Neurology Department, King Fahad Specialist Hospital-Dammam, Khobar, Saudi Arabia; Hospital Universitario de CEMIC, Buenos Aires, Argentina; South Eastern HSC Trust, Belfast, UK; Postgraduate Institute of Medical Education and Research (PGIMER), Chandigarh, India; University MS Centre, Hasselt-Pelt, Belgium Noorderhart Rehabilitation & MS Center, Pelt and Hasselt University, Hasselt, Belgium; AHEPA University Hospital, Thessaloniki, Greece; Rashid Hospital, Dubai, United Arab Emirates; St. Michael’s Hospital, Toronto, ON, Canada; University Hospital Reina Sofia, Cordoba, Spain; Universidade Metropolitana de Santos, Santos, Brazil; Jewish General Hospital, Montreal, QC, Canada; Geelong Hospital, Geelong, VIC, Australia; Booali Sina Hospital, Neurology Department, Faculty of Medicine, Mazandaran University of Medical Sciences, Sari, Iran; St. Vincent’s Hospital, Fitzroy, Melbourne, VIC, Australia; Department of Neurology, Antwerp University Hospital, Edegem, Belgium Translational Neurosciences Research Group, Faculty of Medicine and Health Sciences, University of Antwerp, Antwerp, Belgium; Concord Repatriation General Hospital, Sydney, NSW, Australia; AZ Alma Ziekenhuis, Damme, Belgium; Hospital General Universitario de Alicante, Alicante, Spain; Lyell McEwin Hospital, Elizabeth Vale, SA, Australia; Division of Psychological Medicine and Clinical Neurosciences, Cardiff University School of Medicine, Cardiff, UK Helen Durham Centre for Neuroinflammation, University Hospital of Wales, Cardiff, UK; Department of Clinical Neurosciences, University of Cambridge, Cambridge, UK; MS Centre, Department of Neurology, Royal Melbourne Hospital, Melbourne, VIC, Australia CORe, Department of Medicine, University of Melbourne, Melbourne, VIC, Australia; MS Centre, Department of Neurology, Royal Melbourne Hospital, Melbourne, VIC, Australia CORe, Department of Medicine, University of Melbourne, Melbourne, VIC, Australia Melbourne School of Psychological Sciences, University of Melbourne, Melbourne, VIC, Australia; Department of Clinical Neurosciences, University of Cambridge, Cambridge, UK; MS Centre, Department of Neurology, Royal Melbourne Hospital, Melbourne, VIC, Australia CORe, Department of Medicine, University of Melbourne, Melbourne, VIC, Australia; Department of Clinical Neurosciences, University of Cambridge, Cambridge, UK

**Keywords:** Multiple sclerosis, prognosis

## Abstract

**Background:**

The prognostic significance of non-disabling relapses in people with relapsing-remitting multiple sclerosis (RRMS) is unclear.

**Objective:**

To determine whether early non-disabling relapses predict disability accumulation in RRMS.

**Methods:**

We redefined mild relapses in MSBase as ‘non-disabling’, and moderate or severe relapses as ‘disabling’. We used mixed-effects Cox models to compare 90-day confirmed disability accumulation events in people with exclusively non-disabling relapses within 2 years of RRMS diagnosis to those with no early relapses; and any early disabling relapses. Analyses were stratified by disease-modifying therapy (DMT) efficacy during follow-up.

**Results:**

People who experienced non-disabling relapses within 2 years of RRMS diagnosis accumulated more disability than those with no early relapses if they were untreated (*n* = 285 vs 4717; hazard ratio (HR) = 1.29, 95% confidence interval (CI) = 1.00–1.68) or given platform DMTs (*n* = 1074 vs 7262; HR = 1.33, 95% CI = 1.15–1.54), but not if given high-efficacy DMTs (*n* = 572 vs 3534; HR = 0.90, 95% CI = 0.71–1.13) during follow-up. Differences in disability accumulation between those with early non-disabling relapses and those with early disabling relapses were not confirmed statistically.

**Conclusion:**

This study suggests that early non-disabling relapses are associated with a higher risk of disability accumulation than no early relapses in RRMS. This risk may be mitigated by high-efficacy DMTs. Therefore, non-disabling relapses should be considered when making treatment decisions.

## Introduction

The prognostic significance of non-disabling relapses in people with relapsing-remitting multiple sclerosis (RRMS) is unclear. However, the European Medicines Agency^1,2^ restricts the use of certain disease-modifying therapies (DMTs), particularly natalizumab and fingolimod, to only those with disabling relapses. Whether this is justified remains debated^3^ but has important implications: early initiation of high-efficacy DMTs can mitigate future disability,^4,5^ so if early non-disabling relapses predict disability accumulation, disregarding them risks preventable long-term disability. We aimed to determine whether non-disabling relapses early in RRMS predict disability accumulation.

## Methods

The MSBase registry was approved by the Melbourne Health Human Research Ethics Committee, and by the local ethics committees in participating centres. Written informed consent was obtained from participants as required.

Longitudinal clinical data from 78,531 patients were extracted from MSBase in May 2022. For inclusion, patients required a diagnosis of clinically-definite RRMS, the MSBase minimal dataset (date of birth, MS centre and dates of MS symptom onset, clinical follow-up, relapses and DMT), a baseline disability score (defined below), and, for the non-disabling relapse group, complete relapse severity information for 2 years from RRMS diagnosis – ‘early MS’.

MSBase classifies relapses as ‘mild’ if they do not affect activities of daily living (ADLs); ‘moderate’ if they affect ADLs; and ‘severe’ if they require hospitalisation. For the purposes of this study, ‘non-disabling’ relapses were those graded as mild by clinicians in MSBase and ‘disabling’ relapses were those graded as moderate or severe, in alignment with treatment guidelines.^6^

We compared people with exclusively non-disabling relapses during the 2-year early MS period to: (a) those with no early relapses; and (b) those with at least one early disabling relapse. These relapse severity groups were each stratified by the highest efficacy DMT received: (i) untreated; (ii) only platform DMTs (interferon-beta, glatiramer acetate, dimethyl-fumarate or teriflunomide);^6^ and (iii) high-efficacy DMTs (alemtuzumab, anti-CD20 antibodies, cladribine, daclizumab, haematopoietic stem cell transplantation, mitoxantrone, natalizumab, or sphingosine-1-phospate modulators) at any point from RRMS diagnosis to the end of follow-up.

Baseline disability was defined as the first Expanded Disability Status Scale (EDSS) score within the 2-year early MS period when comparing the non-disabling relapse and no relapse group; or the first EDSS score after the early MS period, when comparing with the disabling relapse group, to exclude disability acquired directly from disabling relapses. In case non-disabling relapses also directly caused disability accumulation, a sensitivity analysis was performed comparing the non-disabling relapse and no relapse groups with the baseline as the first EDSS score after the early MS period. Baseline EDSS scores within 60 days after a relapse were excluded. We used mixed-effects Cox models to compare cumulative hazards of a 90-day confirmed disability accumulation event, defined as an increase in EDSS score of ⩾ 1.0 (or ⩾ 1.5 if the baseline EDSS = 0, or 0.5 if the baseline EDSS ⩾ 5.5), adjusted for age, sex, year of baseline EDSS, interval between the first symptom and RRMS diagnosis, EDSS score at RRMS diagnosis and treatment centre (as a random intercept). When comparing the disabling relapse and non-disabling relapse groups, the models were also adjusted for the number of early MS relapses. The Schoenfeld^7^ global test was used to detect violation of the proportional hazards assumption. Statistical analysis was performed using R version 4.1.3.

## Results

The characteristics of the included patients (Supplemental Figure 1) are outlined in Table 1.

**Table 1.** Characteristics of the studied populations.

Analyses comparing people with no early relapses to those with early non-disabling relapses						
	Untreated		Platform DMT		High-efficacy DMT	
	No relapse	Non-disabling	No relapse	Non-disabling	No relapse	Non-disabling
n	4717	285	7262	1074	3534	572
Female (number, %)	3359 (71.2%)	214 (75.1%)	5096 (70.2%)	785 (73.1%)	2479 (70.1%)	418 (73.1%)
Age, years (mean, IQR)	35 (27–43)	31 (26–41)	34 (27–42)	31 (25–38)	32 (26–40)	29 (23–35)
Baseline EDSS (median, IQR)	1.5 (1.0–2.5)	1.5 (1.0–2.0)	1.5 (1.0–2.5)	1.5 (1.0–2.0)	2.0 (1.0–3.0)	2.0 (1.0–2.5)
Year of inclusion (median, IQR)	2012 (2007–2016)	2011 (2006–2015)	2012 (2008–2016)	2011 (2007–2014)	2014 (2011–2017)	2012 (2009–2015)
Duration of MS at inclusion, years (median, IQR)	1.25 (0.31–4.33)	1.00 (0.28–3.68)	1.00 (0.26–3.28)	0.82 (0.25–2.57)	0.70 (0.23–2.50)	0.62 (0.25–1.77)
Follow-up duration (baseline to last recorded EDSS), years (median, IQR)	1.02 (0.27–4.59)	2.91 (0.85–7.87)	4.44 (1.69–8.55)	6.20 (3.03–10.40)	4.82 (2.31–8.20)	7.09 (4.33–9.85)
Proportion of follow-up on DMT (median, IQR)	N/A	N/A	91% (69%–99%)	84% (63%–95%)	91% (76%–97%)	89% (75%–96%)
Analyses comparing people with early disabling relapses to those with early non-disabling relapses						
	Untreated		Platform DMT		High–efficacy DMT	
	Disabling	Non–disabling	Disabling	Non-disabling	Disabling	Non-disabling
n	333	192	1510	925	1631	595
Female (number, %)	251 (75.4%)	151 (78.6%)	1092 (72.3%)	675 (73.0%)	1165 (71.4%)	432 (72.6%)
Age, years (mean, IQR)	36 (28–45)	34 (27–43)	34 (28–41)	34 (27–41)	32 (26–39)	32 (26–39)
Baseline EDSS (median, IQR)	2.0 (1.0–3.5)	1.5 (1.0–2.0)	2.0 (1.0–3.0)	1.5 (1.0–2.0)	2.0 (1.0–3.0)	1.5 (1.0–2.0)
Year of inclusion (median, IQR)	2012 (2006–2016)	2012 (2007–2016)	2011 (2007–2015)	2012 (2008–2016)	2014 (2011–2017)	2014 (2011–2017)
Duration of MS at inclusion, years (median, IQR)	2.82 (2.19–5.20)	2.93 (2.25–5.38)	2.90 (2.24–5.07)	2.63 (2.17–4.16)	2.54 (2.16–3.99)	2.57 (2.16–3.78)
Follow-up duration (baseline to last recorded EDSS), years (median, IQR)	3.75 (1.16–8.38)	3.45 (1.00–8.84)	6.05 (2.28–10.6)	5.22 (2.35–9.09)	5.00 (2.42–8.26)	5.40 (2.91–7.93)
Proportion of follow-up on DMT (median, IQR)	N/A	N/A	100% (96%–100%)	100% (98%–100%)	99% (90%–100%)	99% (89%–100%)
Number of early relapses (median, IQR)	4 (3–6)	3 (2–5)	4 (3–6)	3 (3–5)	4 (3–6)	3 (2–5)

DMT = disease-modifying therapy; EDSS = Expanded Disability Status Scale; IQR = interquartile range; N/A = not applicable; RRMS = relapsing-remitting multiple sclerosis. ‘Early’ relapses are those in the first 2 years after RRMS diagnosis.

**Figure 1.**
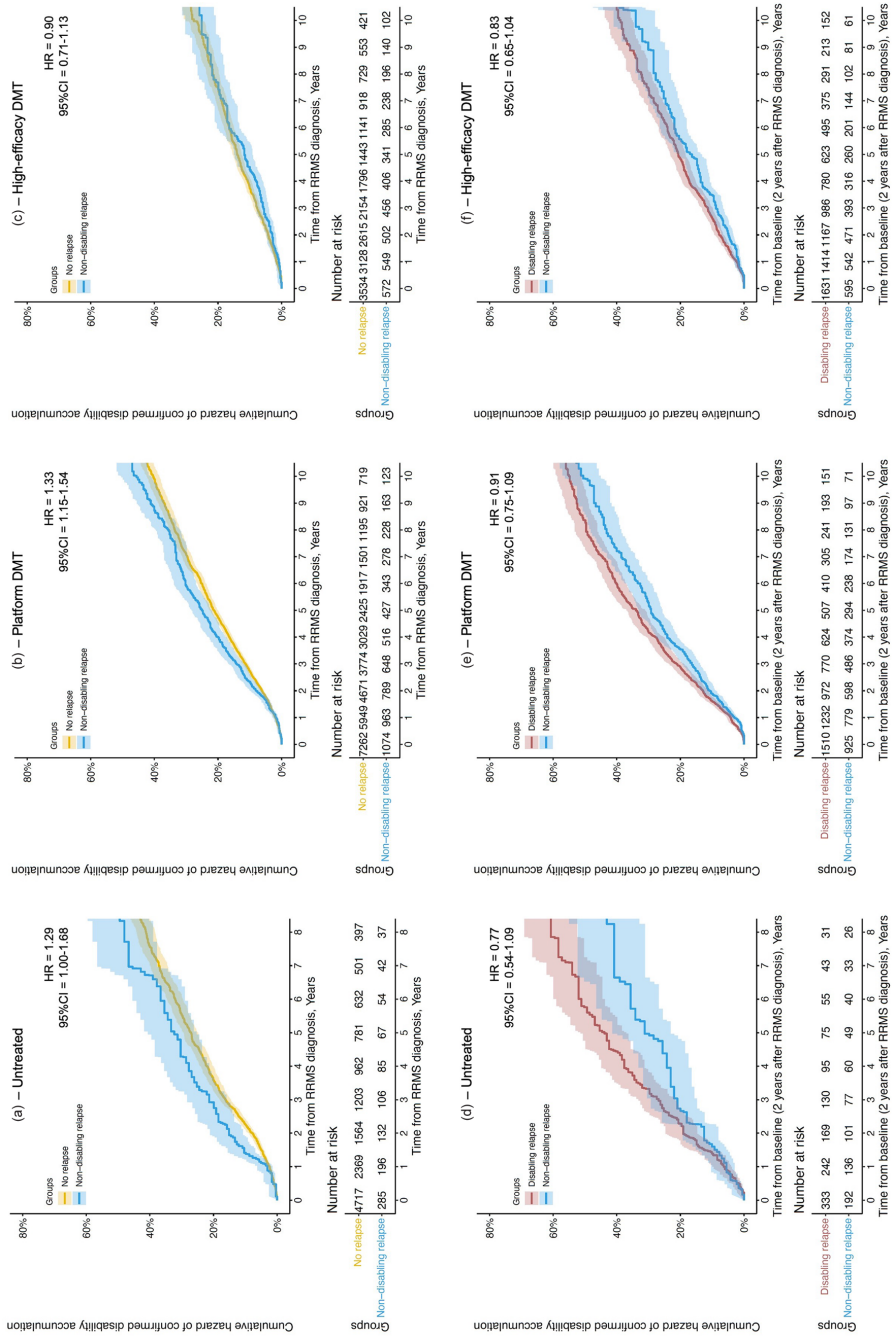
Comparison of the cumulative hazard of a 90-day confirmed disability accumulation event between people with exclusively non-disabling relapses and no relapses (a–c), or exclusively non-disabling relapses and at least one disabling relapse (d–f), in the 2 years after RRMS diagnosis, in people who remained untreated throughout follow-up (a and d), were given only platform DMTs (b and e) or were given high-efficacy DMTs (c and f) at any point during follow-up. DMT = disease-modifying therapy; HR = hazard ratio; 95% CI = 95% confidence interval; RRMS = relapsing-remitting multiple sclerosis.

People who exclusively experienced non-disabling relapses in the 2-year early MS period accumulated more disability than those with no early relapses if they remained untreated (*n* = 285 vs 4717; hazard ratio [HR] = 1.29, 95% confidence interval [CI] = 1.00–1.68), or received only platform DMTs (*n* = 1074 vs 7262; HR = 1.33, 95% CI = 1.15–1.54), but not if they received high-efficacy DMTs (n = 572 vs 3534; HR = 0.90, 95% CI = 0.71–1.13; Figure 1(a)–(c)). These results were similar in a sensitivity analysis with the baseline moved after the early MS period, thus excluding that incomplete recovery from early non-disabling relapses was solely responsible for the observed differences in disability: untreated (*n* = 192 vs 2449; HR = 1.23; 95% CI = 0.90–1.68); platform DMTs (n = 925 vs 6112; HR = 1.20, 95% CI = 1.03–1.40); high-efficacy DMTs (n = 595 vs 3622; HR = 0.97, 95% CI = 0.78–1.21).

Differences in disability accumulation between people who exclusively experienced non-disabling relapses in the early MS period and those who experienced any disabling relapses in early MS did not reach statistical significance (Figure 1(d)–(f)): untreated (*n* = 192 vs 333; HR = 0.77; 95% CI = 0.54–1.09); platform DMTs (n = 925 vs 1510; HR = 0.91, 95% CI = 0.75–1.09); high-efficacy DMTs (*n* = 595 vs 1631; HR = 0.83, 95% CI = 0.65–1.04).

## Discussion

In this international observational study, people with RRMS who experienced early non-disabling relapses had a higher risk of disability accumulation than those with no early relapses, if they were untreated or received only platform DMTs during follow-up. However, this association was not observed in people who received high-efficacy DMTs. This suggests, contrary to current guidance,^1,2^ that non-disabling relapses should be considered in decisions to initiate or escalate treatment, including with high-efficacy therapies.

We did not confirm statistically whether distinguishing early relapse severity has prognostic significance, but the power of these analyses was limited by smaller numbers. There was a non-significant trend towards more disability accumulation with early disabling relapses over early non-disabling relapses, which was mitigated with DMTs. This might be explained by previous observations that relapse phenotypes tend to recur,^8^ and incomplete recovery from the first relapse, which is more common for disabling relapses, predicts incomplete recovery from subsequent relapses.^9^

### Limitations

Relapse severity was non-standardised. However, the three-category classification is defined by the MSBase Study Protocol and reflects current DMT-prescribing restrictions^6^ as applied in real-world clinical practice. By including treatment centre in our models, we mitigated variation among centres in relapse severity classification. Data were missing on the severity of a large proportion of early relapses, which may have limited both our power to detect differences between disabling relapses and non-disabling relapses and the generalisability of our conclusions. We also did not perform a direct comparison between matched groups of patients treated with different DMT efficacies following a non-disabling relapse. In view of these limitations, further studies are required to confirm the futility of making treatment decisions based on relapse severity.

The follow-up duration in the untreated populations was short (median 1.02–3.75 years). However, a similar risk of disability was observed in patients with non-disabling relapses treated with platform DMTs over a longer follow-up (median 4.44–6.20 years).

On-treatment relapses may be associated with worse outcomes than off-treatment relapses,^10^ but we did not explore this here. We also did not explore the prognostic value of radiological disease activity.

## Conclusion

This study suggests that people with early non-disabling relapses have a higher risk of disability accumulation than those with no early relapses and that this risk might be mitigated by high-efficacy DMTs. Therefore, even non-disabling relapses should be considered when making treatment decisions.

## Supplemental Material

sj-docx-1-msj-10.1177_13524585231151951 – Supplemental material for Early non-disabling relapses are important predictors of disability accumulation in people with relapsing-remitting multiple sclerosis Click here for additional data file. Supplemental material, sj-docx-1-msj-10.1177_13524585231151951 for Early non-disabling relapses are important predictors of disability accumulation in people with relapsing-remitting multiple sclerosis by Cyrus Daruwalla, Vahid Shaygannejad, Serkan Ozakbas, Eva Kubala Havrdova, Dana Horakova, Raed Alroughani, Cavit Boz, Francesco Patti, Marco Onofrj, Alessandra Lugaresi, Sara Eichau, Marc Girard, Alexandre Prat, Pierre Duquette, Bassem Yamout, Samia J Khoury, Seyed Aidin Sajedi, Recai Turkoglu, Ayse Altintas, Olga Skibina, Katherine Buzzard, Pierre Grammond, Rana Karabudak, Anneke van der Walt, Helmut Butzkueven, Davide Maimone, Jeannette Lechner-Scott, Aysun Soysal, Nevin John, Julie Prevost, Daniele Spitaleri, Cristina Ramo-Tello, Oliver Gerlach, Gerardo Iuliano, Matteo Foschi, Radek Ampapa, Vincent van Pesch, Michael Barnett, Nevin Shalaby, Marie D’hooghe, Jens Kuhle, Maria Jose Sa, Marzena Fabis-Pedrini, Allan Kermode, Saloua Mrabet, Riadh Gouider, Suzanne Hodgkinson, Guy Laureys, Liesbeth Van Hijfte, Richard Macdonell, Celia Oreja-Guevara, Edgardo Cristiano, Pamela McCombe, Jose Luis Sanchez-Menoyo, Bhim Singhal, Yolanda Blanco, Stella Hughes, Justin Garber, Claudio Solaro, Chris McGuigan, Bruce Taylor, Koen de Gans, Mario Habek, Abdullah Al-Asmi, Simu Mihaela, Tamara Castillo Triviño, Talal Al-Harbi, Juan Ignacio Rojas, Orla Gray, Dheeraj Khurana, Bart Van Wijmeersch, Nikolaos Grigoriadis, Jihad Inshasi, Jiwon Oh, Eduardo Aguera-Morales, Yara Fragoso, Fraser Moore, Cameron Shaw, Seyed Mohammad Baghbanian, Neil Shuey, Barbara Willekens, Todd A Hardy, Danny Decoo, Angel Perez sempere, Deborah Field, Ray Wynford-Thomas, Nick G Cunniffe, Izanne Roos, Charles B Malpas, Alasdair J Coles, Tomas Kalincik and J William L Brown in Multiple Sclerosis Journal
