# Supplementary material for: Early non-disabling relapses are important predictors of disability accumulation in people with relapsing-remitting multiple sclerosis
Source: Mult Scler. 2023 Feb 27;29(7):875–83. doi: 10.1177/13524585231151951 (PMC10278381; doi:10.1177/13524585231151951)

**Supplementary figure 1:** The study cohort

Abbreviations: EDSS = expanded disability status scale; RRMS = relapsing-remitting multiple sclerosis


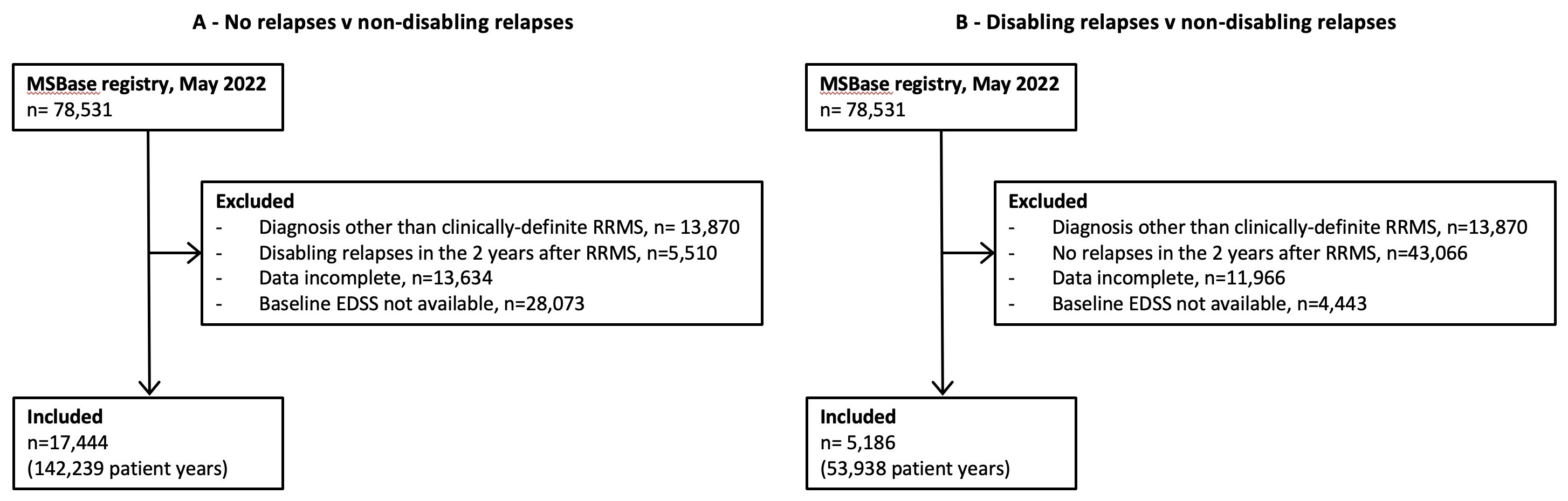

Supplement: sj-docx-1-msj-10.1177_13524585231151951 – Supplemental material for Early non-disabling relapses are important predictors of disability accumulation in people with relapsing-remitting multiple sclerosis [file sj-docx-1-msj-10.1177_13524585231151951.docx]
